# Findings and lessons from establishing Zika virus surveillance in southern Viet Nam, 2016

**DOI:** 10.5365/wpsar.2018.9.2.014

**Published:** 2019-05-14

**Authors:** Lan Trong Phan, Quang Chan Luong, Thi Hong Hien Do, Cindy H Chiu, Thang Minh Cao, Thao Thi Thanh Nguyen, Hai Thanh Diep, Thao Phuong Huynh, Dung Tri Nguyen, Nga Hong Le, Satoko Otsu, Phu Dac Tran, Thuong Vu Nguyen, Masaya Kato

**Affiliations:** a Pasteur Institute, Ho Chi Minh City, Viet Nam.; b World Health Organization Viet Nam Country Office, Viet Nam.; c Tohoku University Graduate School of Medicine, Japan.; d Preventive Medicine Centre, Ho Chi Minh City, Viet Nam.; e General Department of Preventive Medicine, Ministry of Health, Viet Nam.; f World Health Organization Regional Office for the Western Pacific, Philippines.

## Abstract

**Objective:**

To document the evolution and optimization of the Zika virus (ZIKV) disease surveillance system in southern Viet Nam in 2016 and to describe the characteristics of the identified ZIKV-positive cases.

**Methods:**

We established a sentinel surveillance system to monitor ZIKV transmission in eight sites in eight provinces and expanded the system to 71 sites in 20 provinces in southern Viet Nam in 2016. Blood and urine samples from patients who met the case definition at the sentinel sites were tested for ZIKV using real-time reverse transcription polymerase chain reaction at the Pasteur Institute in Ho Chi Minh City (PI-HCMC). We conducted descriptive analysis and mapped the ZIKV-positive cases.

**Results:**

In 2016, 2190 specimens from 20 provinces in southern Viet Nam were tested for ZIKV at PI-HCMC; 626 (28.6%), 484 (22.1%), 35 (1.6%) and 1045 (47.7%) tests were conducted in the first, second, third and fourth quarters of the year, respectively. Of these tested specimens, 214 (9.8%) were ZIKV positive with 212 (99.1%) identified in the fourth quarter. In the fourth quarter, the highest positivity rate was those in age groups 30–39 years (30.0%) and 40–59 years (31.6%). Of the 214 ZIKV-positive patients, 210 (98.1%) presented with rash, 194 (90.7%) with fever, 149 (69.6%) with muscle pain, 123 (57.5%) with joint pain and 66 (30.8%) with conjunctivitis.

**Discussion:**

The surveillance system for ZIKV disease underwent several phases of optimization in 2016, guided by the most up-to-date local data. Here we demonstrate an adaptable surveillance system that detected ZIKV-positive cases in southern Viet Nam.

## Objective

In Viet Nam, although Zika virus (ZIKV) disease was not listed as a nationally notifiable disease before 2016, previous literature suggests that it is not a new disease in the country. There has been evidence of possible transmission of ZIKV disease dating as far back as 1954 when neutralizing antibodies against ZIKV were detected in the indigenous population in northern Viet Nam. (*1*) More recently, a study that retrospectively tested 5617 dengue-negative serum samples collected at seven hospital outpatient departments in 2010–2014 in southern Viet Nam also identified ZIKV-positive cases from 2013. (*2*) When Zika disease was declared by the World Health Organization (WHO) as a public health emergency of international concern (PHEIC) in 2016, (*3*) several ZIKV-positive cases of travellers who visited Viet Nam before symptom onset were reported. (*4*-*6*)

**Fig. 1:**
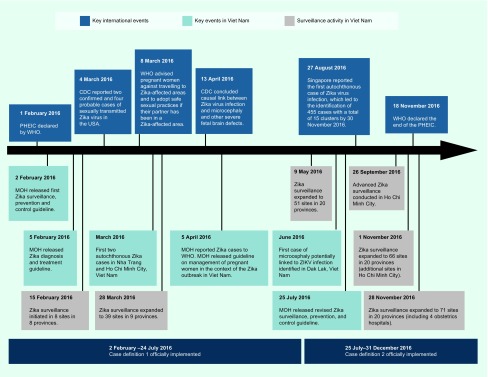
Timeline of key events related to the ZIKV outbreak internationally and in Viet Nam, 2016

CDC, Centers for Disease Control and Prevention (United States); PHEIC, public health emergency of international concern; WHO, World Health Organization; USA, United States of America; health ministry, Ministry of Health (Viet Nam); ZIKV, Zika virus.

In response to WHO’s declaration of PHEIC, Viet Nam responded swiftly in rolling out Zika surveillance, prevention and control guidelines on 2 February 2016 and the Zika diagnosis and treatment guidelines on 5 February 2016 (see **Fig. 1**). (*7*, *8*) In March 2016, two cases of autochthonous transmission of ZIKV infection were detected in Nha Trang and Ho Chi Minh City (HCMC) and reported to WHO. (*9*) The case in HCMC was a pregnant woman who had fetal demise at week nine of her pregnancy. (*10*) In June 2016, an infant, born in Dak Lak, Viet Nam, (*11*) was the first and only known case of microcephaly potentially linked to ZIKV infection in Viet Nam. The ZIKV disease surveillance data are critical to better understand the local outbreak and to guide the appropriate level of response. As with any new surveillance system, adjustments may be needed as part of the optimization process. Surveillance for ZIKV infection was initially based on existing dengue virus disease surveillance; however, given the largely asymptomatic and mild clinical presentation of ZIKV disease, an independent surveillance strategy specifically tailored for ZIKV disease was needed. As the local and international situations evolved, Viet Nam also adjusted its surveillance approach and expanded the number of ZIKV disease surveillance sites in southern Viet Nam where the majority of the cases occurred.

The objectives of this paper were to document the evolution and optimization of the ZIKV disease surveillance system in southern Viet Nam in 2016 and to describe the characteristics of the ZIKV-positive cases identified through the surveillance system. We hope that by sharing our lessons we can highlight the practical realities of implementing a new surveillance system for a re-emerging disease in the context of a rapidly evolving international public health emergency and a local ZIKV disease outbreak.

## Methods

### Epidemiological surveillance

#### Surveillance sites

A sentinel surveillance system to monitor ZIKV transmission in southern Viet Nam was established using a phased approach in 2016 (**Fig. 1**). Sentinel sites were gradually expanded from an initial eight sites in eight provinces in February 2016 to 71 sites in 20 provinces by November 2016 (**Fig. 1**). In phase I, the surveillance system was first established using the existing dengue sentinel surveillance system. We targeted eight southern provinces deemed as high-risk areas for active transmission of Zika with the assumption that high-risk areas for dengue transmission would also be high-risk areas for Zika transmission. High-risk areas were selected based on three factors based on the guidance from the Ministry of Health and National Program for Dengue Control: 1) the epidemiology of dengue fever (> 100 dengue cases per 100 000 population); 2) the *Aedes aegypti* mosquito density index (> 0.5 female mosquito per house per day); and 3) the tourist flow (presence of international and domestic transportation and famous sites frequented by tourists). In each of the eight selected provinces, one existing surveillance site at either a provincial or district hospital was selected and began case finding from 15 February 2016 (**Fig. 1**). One month later, in phase II, sentinel surveillance was rolled out in HCMC, the largest city in southern Viet Nam. The roll-out included all district hospitals in the city (**Fig. 1**). From May 2016, in phase III, the remaining 12 provinces in southern Viet Nam also began implementing the sentinel ZIKV surveillance system in at least one district hospital per province. By November 2016, when ZIKV-positive cases peaked in HCMC, the ZIKV surveillance system had been further expanded to all four city obstetrics hospitals (**Fig. 1**). In addition, eight commune health stations and 15 private clinics participated in the sentinel surveillance system in the fourth quarter of 2016.

#### Case definitions

All outpatients meeting the case definition at each sentinel hospital were included in our analysis. In 2016, the Viet Nam Ministry of Health issued an initial and a revised official case definitions guided by the international outbreak situation and local data from Viet Nam, as listed in **Table 1**.

**Table 1:** ZIKV surveillance case definitions used in Viet Nam, February–December 2016

Case definition 1 Date of national guideline implemented: 2 February 2016
**Suspected case** Any patient presenting with fever, rash AND at least one of the following: • Conjunctivitis • Joint pain, muscle pain • Headache AND • Travel in/to/from a Zika-affected area within 12 days before symptom onset. **Confirmed case** Suspected cases confirmed with ZIKV infection by laboratory diagnostic tests including molecular biology technique, virus isolation, or serology.
**Case definition 2** **Date of national guideline implemented: 25 July 2016**
**Suspected case** Any patient presenting with rash AND at least two of the following symptoms: • Fever ≤ 38.5 °C • Non-purulent conjunctivitis • Joint pain, swelling around the joints • Muscle pain. **Probable case** Any patient who meets the criteria for a suspected case AND has Zika IgM antibodies, with no evidence of infection with other flaviviruses. **Confirmed case** Any patient who meets the criteria for a suspected or probable case AND has laboratory confirmation of recent ZIKV infection through: • Culture of ZIKV isolates, or • Identification of the specific gene fragment of ZIKV by molecular biology technique, or • Detection of Zika IgM antibodies AND plaque reduction neutralization (PRNT90) for ZIKV with titres ≥ 20 and ZIKV titre ratio ≥ 4 compared to other flaviviruses.

ZIKV, Zika virus.

#### Case investigation

We conducted case investigations and interviewed the patients who met the case definition using a one-page, semi-structured questionnaire to obtain and confirm information on their socio-demographic characteristics, signs and symptoms, dates of symptom onset and travel histories.

### Laboratory testing

We collected blood and urine samples from patients who met the case definition. On the same day, we sent the specimens to the Pasteur Institute in HCMC (PI-HCMC) where testing was conducted once per week for ZIKV. A patient was defined as a ZIKV-positive case when ZIKV was detected using real-time reverse transcription polymerase chain reaction (RT–PCR) with Trioplex reagents provided by the United States Centers for Disease Control and Prevention. The ZIKV testing procedure was developed in accordance with the primer and probe sequences as described previously by Lanciotti et al. (*12*)

### Data collection and analysis

Questionnaire data and test results were first collected on paper forms and later entered and analysed in Microsoft Excel. We conducted descriptive analysis to examine the data by person, place and time. Spot mapping was conducted using ArcGIS (ESRI, Redlands, CA, USA) to look at the geographical spread of ZIKV-positive cases over time.

### Ethical approval

The data presented in this manuscript were for public health surveillance (*13*) and not research; therefore, approval from an ethics committee was not sought.

## Results

**Fig. 2:**
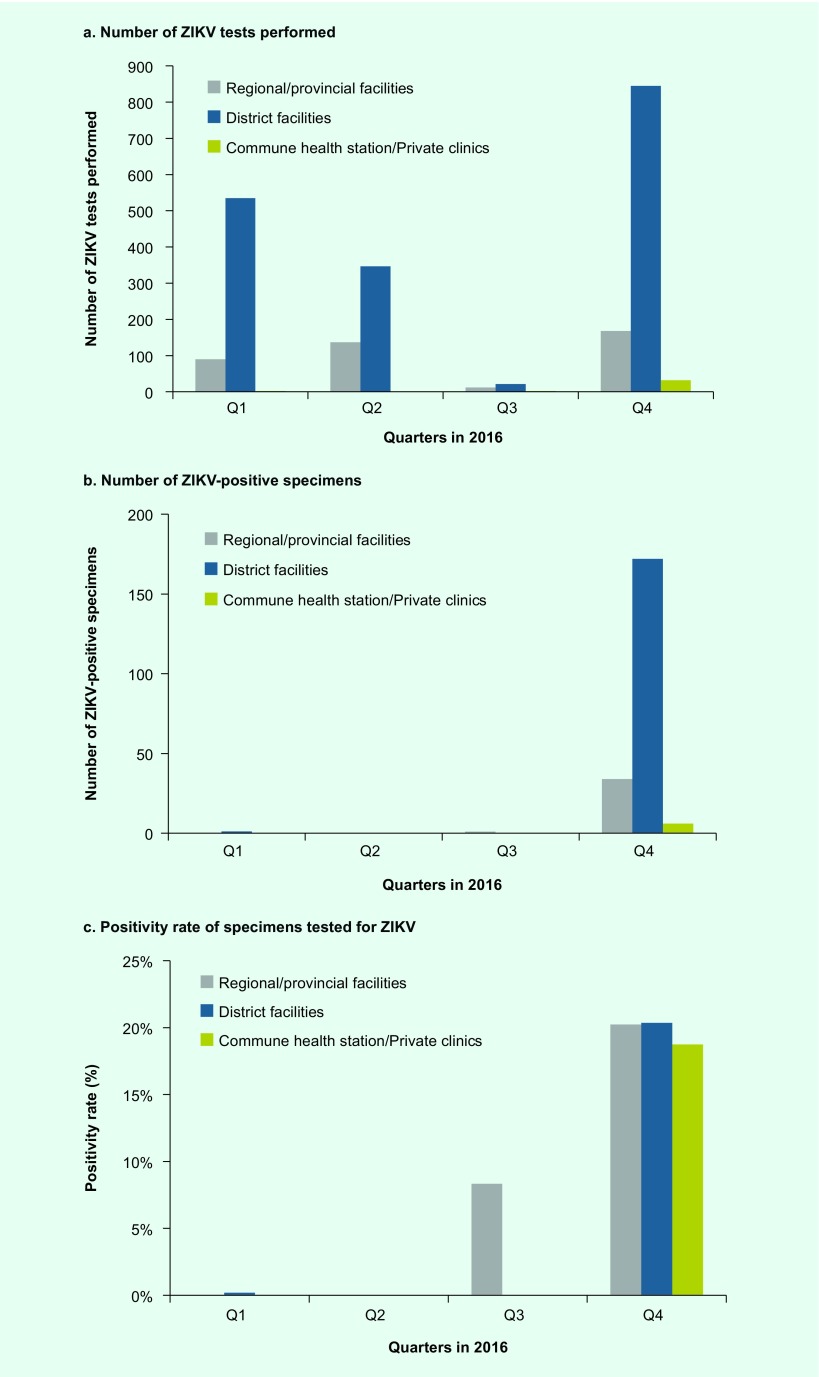
Number of ZIKV tests performed, number of specimens tested positive, and positivity rate by administrative level of facility, Southern Viet Nam, 2016

In total, 2190 specimens from 20 provinces in southern Viet Nam were tested for ZIKV at PI-HCMC in 2016; 626 (28.6%) tests were performed in quarter 1; 484 (22.1%) tests in quarter 2; 35 (1.6%) tests in quarter 3; and 1045 (47.7%) tests in quarter 4. Two distinct waves of ZIKV-positive cases occurred in 2016 (**Fig. 2**). A total of 214 (9.8%) of the 2190 tested specimens were positive for ZIKV. Of the 214 ZIKV-positive specimens, the majority were identified in quarter 4 (*n* = 212, 99.1%). The positivity rate was 0.2% in quarter 1 (1 positive among 626 samples tested), 0% in quarter 2 (0 positive among 484 samples tested), 2.9% in quarter 3 (1 positive among 35 samples tested) and 20.3% in quarter 4 (212 positive among 1045 samples tested).

ZIKV, Zika virus.

All ZIKV tests were performed at the Pasteur Institute in HCMC. The regional and provincial facilities include infectious disease hospitals, obstetrics and gynaecology hospitals, paediatric hospitals, provincial general hospitals, and provincial preventive medicine centres. The district facilities include district hospitals.

Most (*n* = 1749; 79.9%) of the specimens were collected at district hospitals where 173 (9.9%) tested positive; 407 (18.6%) specimens were collected at regional and provincial facilities where 35 (8.6%) specimens tested positive for ZIKV. These facilities included infectious disease hospitals, obstetrics and gynaecology hospitals, paediatric hospitals, provincial general hospitals and provincial preventive medicine centres. During the quarter with the highest incidence (quarter 4), a small number (*n* = 32; 1.5%) of specimens were obtained from one commune health station (*n* = 5; 0.2%) and private clinics (*n* = 27; 1.2%) in one of the sentinel districts (District 2); of these 32 specimens, 6 (18.8%) tested positive (1 [3.1%] from commune health stations and 5 [15.6%] from private clinics.) The positivity rate in quarter 4 was similar across levels of facilities: 20.2% at regional and provincial facilities, 20.4% at district facilities and 18.8% at commune health station and private clinics (**Fig. 2**).

The characteristics of the individuals tested for ZIKV and those tested positive are summarized in **Table 2**. Pregnant women had relatively high positivity rates compared to others tested during the year (22.9%) and in quarter 4 specifically (24.6%). In children and non-pregnant women, the positivity rate in quarter 4 was more than double that of the year as a whole. The positivity rates in non-pregnant women and men were similar (8.7% versus 8.0% in the whole year; 18.8% versus 20.2% in quarter 4). Those in age groups 30–39 and 40–59 had the highest positivity rate at 16.5% and 14.8% in the whole year and 30.0% and 31.6% in quarter 4, respectively. Fewer children under 15 years of age were tested than adults, and the positivity rate for children was 0.7% during the whole year and 3.4% in quarter 4. Of the 145 children aged 5–9 years old who were tested, none tested positive for ZIKV.

**Table 2:** Demographics of individuals tested for ZIKV and those with laboratory-confirmed ZIKV infection, southern Viet Nam, 2016

-	Quarters 1 – 4			Quarter 4		
	Number tested	Number positive	Percentage positive (%)	Number tested	Number positive	Percentage positive (%)
**Population group**						
Pregnant women	205	47	22.9	187	46	24.6
Other women	1143	100	8.7	526	99	18.8
Men	842	67	8.0	332	67	20.2
Children (< 15 years old)	280	2	0.7	59	2	3.4
**Age (years)**						
< 5	135	2	1.5	28	2	7.1
5–9	145	0	0	31	0	0
10–19	350	27	7.7	147	27	18.4
20–29	844	79	9.4	474	78	16.5
30–39	417	69	16.5	227	68	30.0
40–59	209	31	14.8	98	31	31.6
60+	65	6	9.2	26	6	23.1
Unknown	25	0	0	14	0	0
**Location of detection**						
Ho Chi Minh City	1422	194	13.6	788	192	24.4
Other provinces	716	20	2.8	232	20	8.6
Unknown	52	0	0	25	0	0
**Total**	**2190**	**214**	**9.8**	**1045**	**212**	**20.3**

ZIKV, Zika virus.

**Fig. 3:**
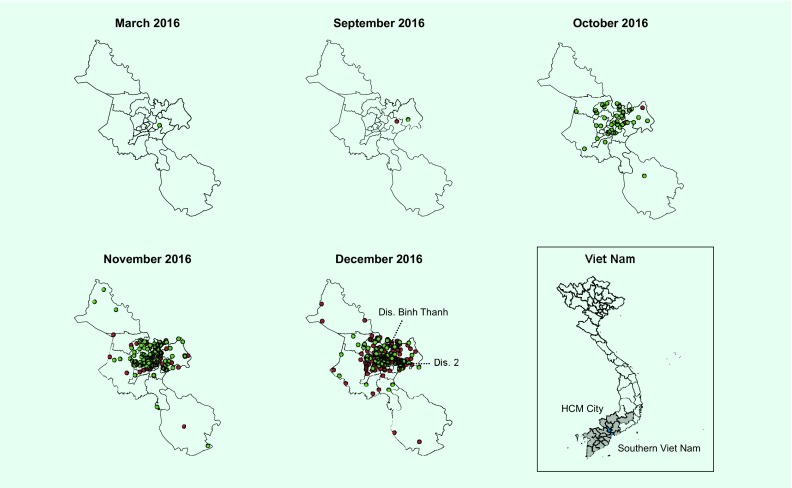
Spot map of confirmed ZIKV-positive cases identified through the ZIKV surveillance system, Ho Chi Minh City, Southern Viet Nam, 2016

The positivity rate was higher in HCMC than in other provinces in southern Viet Nam in 2016 (13.6% versus 2.8%) and in quarter 4 (24.4% versus 8.6%) (**Table 2**). Within HCMC, the positive cases were concentrated in certain districts (**Fig. 3**): Binh Thanh District, District 2 and District 12 had the highest number of positive cases detected in 2016 and in quarter 4.

Dis., district; HCM, Ho Chi Minh; ZIKV, Zika virus.

Green spots: cases of the current month; Red spots: cases from the start of the outbreak in February 2016 to the previous month.

A patient can often present with multiple symptoms. Of the 214 patients who were positive for ZIKV infection, 210 (98.1%) presented with rash, 194 (90.7%) with fever, 149 (69.6%) with muscle pain, 123 (57.5%) with joint pain and 66 (30.8%) with conjunctivitis (data not shown).

## Discussion

In this article, we presented initial efforts to roll out and optimize the surveillance system for ZIKV disease in southern Viet Nam in 2016 and described our early surveillance data. The surveillance system for ZIKV disease underwent several phases of optimization in 2016, guided by the most up-to-date local data. In phase I, PI-HCMC had initially explored integrating ZIKV testing with the existing dengue surveillance. Inpatients with clinically suspected dengue who tested negative for dengue by nonstructural protein 1 or viral culture in 2015 were tested for ZIKV using RT–PCR. All 96 dengue-negative patients were negative for ZIKV (unpublished data). Based on this data, in phase II we decided to implement a dedicated surveillance system for ZIKV that focused on the outpatient ward where patients with milder clinical presentations seek medical care. We believed that this approach was more appropriate than integrating ZIKV surveillance into existing dengue surveillance that is primarily focused on more severe cases from inpatient wards. Focusing on mild cases was also supported by evidence that everyone diagnosed with ZIKV disease outside of Viet Nam after travelling to the country had only mild symptoms. (*4*-*6*) In phase III, after successfully detecting ZIKV-positive cases in outpatient departments, particularly at district hospitals, ZIKV surveillance was gradually expanded throughout southern Viet Nam, focusing on outpatient services at district hospitals in HCMC. In addition, the surveillance case definition was adjusted based on our initial analysis of symptoms associated with ZIKV-positive patients, and guided by the latest literature, which showed a high prevalence of rash in ZIKV-positive patients relative to fever, joint pain, muscle pain and conjunctivitis. (*14*) Our approaches were in line with those proposed by the *Asia Pacific Strategy for Emerging Diseases and Public Health Emergencies*, (*15*) which suggests surveillance systems should be effective, efficient, flexible and promptly adaptable to new available information and needs before, during and after events. In the rapidly changing contexts of an ongoing outbreak, Viet Nam demonstrated flexibility by shifting sampling sites from inpatient to outpatient services and revising case definitions as new knowledge became available. Here, we demonstrate that the surveillance system established during the event in 2016 successfully detected ZIKV-positive cases in southern Viet Nam.

The number of ZIKV-positive results increased markedly in quarter 4, likely reflecting an actual increase in ZIKV transmission during this period. However, the increase could also be due to other contributing factors, including those that may have enhanced the ability of the surveillance system to more efficiently detect ZIKV infections. First, since fewer specimens were tested in quarter 3 due to resource constraints, ZIKV infections may have been underdetected in that quarter, leading to a more dramatic increase in ZIKV infections in quarter 4. Second, the number of surveillance sites doubled from 32 in March to 71 in November, alongside a gradual decentralization of surveillance sites, which resulted in a higher proportion of specimens being collected from district facilities. Third, a new case definition with rash being the primary symptom was first introduced in some district hospitals in March 2016 and was formally implemented across all surveillance sites from August 2016 after accumulating evidence suggested that this could be a better case definition to identify more ZIKV-positive cases. Evolution of the surveillance system may have led to increased efficiency in detecting ZIKV-positive cases.

Data from quarter 4 showed that the positivity rate was similar across regional, provincial and district-level facilities. This result suggests that people with ZIKV infection are attending various types of health facilities, and our ability to detect ZIKV infection might be similar across different levels of health facilities. In one of the sentinel districts (District 2), sample collection was experimentally decentralized to the commune health station and private clinics in quarter 4. The decentralized sample collection had a similar positivity rate of ZIKV infection. However, the large majority of cases in 2016 in HCMC were from district hospitals. Therefore, we expect that district-level facilities will continue to play a central role in the surveillance system for ZIKV disease in southern Viet Nam.

Our data suggested the number of the reported cases differed considerably among districts and the reported cases concentrated in certain districts in HCMC. Careful interpretation is needed to understand such results since several factors could influence the level of case detection to varying degrees. It would be reasonable to consider that there were clusters of active transmission in certain districts. However, the geographical differences could also be due to other factors such as the selection of sentinel sites and the case definition not being applied consistently across hospitals by different clinicians. In addition, intensified guidance from national and local authorities following the ZIKV disease outbreak in Singapore in September 2016 may have raised clinicians’ awareness levels and made it more likely for them to collect and test specimens for ZIKV infections.

Our results showed that adults, especially those aged 30–59 years, were more affected by ZIKV infection than children. This finding is consistent with previous literature that suggests that children with ZIKV infections generally experience mild symptoms. (*16*) However, our results contrast with the age distribution of dengue infection, showing that children are more affected by dengue than adults in southern Viet Nam. (*17*) One possible explanation is that the adult population of southern Viet Nam may have largely developed immunity against dengue, which has been hypothesized to enhance ZIKV infection through antibody-dependent enhancement. (*18*) Ongoing ZIKV seroprevalence surveys in southern Viet Nam may provide a better understanding of population immunity against ZIKV.

The first year of ZIKV surveillance in southern Viet Nam provided critical evidence that will inform surveillance and response efforts in Viet Nam and other countries, and offered important lessons in optimizing ZIKV surveillance systems. Viet Nam’s approach of focusing on outpatient services of health care facilities effectively detected ZIKV-positive cases. However, both the fluctuation in the number of tests performed and the change in case definition made it challenging to interpret trends in local transmission. In addition, given that the ZIKV-positive cases were identified based on the symptoms listed in the case definition, the symptoms of the ZIKV-positive cases shown here may not be representative of all ZIKV-infected individuals.

Moving forward, it may be necessary to prioritize the surveillance approach based on resource availability, especially given the high cost of the current molecular testing methodology using ZIKV PCR. Based on our data, we believe there may be two future directions for ZIKV surveillance to achieve two separate but interrelated objectives. First, Viet Nam may consider monitoring transmission trends by establishing sentinel sites to detect all individuals who meet the case definition, irrespective of age and sex. Based on the system described here, which was developed in response to WHO’s PHEIC declaration for Zika, the Ministry of Health of Viet Nam developed guidelines in 2017 for an ongoing, integrated Chikungunya-Dengue-Zika (CDZ) sentinel surveillance system. (*19*) Second, depending on resource availability, it is important to prioritize Zika testing and surveillance for pregnant women presenting with symptoms consistent with the case definition, regardless of whether they are from sentinel sites or other health facilities to better detect pregnancies that are at risk for microcephaly. In 2017, Viet Nam developed guidelines for all obstetric clinics and hospitals to register, investigate and report pregnancy courses and outcomes of mothers with confirmed ZIKV and babies with microcephaly; (*20*) however, fewer laboratory samples than expected have been collected and tested for ZIKV to date. Therefore, it would be pertinent to continue to strengthen the implementation of the guidelines. In addition to the ZIKV surveillance activities, we believe full genome sequencing of selected specimens in southern Viet Nam may also shed light on the phylogenetic lineage of circulating ZIKV in the country.

## Conclusions

In our interconnected world, all countries are becoming increasingly aware of the borderless nature of emerging and re-emerging infectious diseases. Monitoring a new disease in the population requires establishing a surveillance system in the context of many unknowns while ensuring flexibility of surveillance systems to adapt to changing information and needs. Here, we demonstrate an adaptable sentinel surveillance system for ZIKV disease in Viet Nam, where it was optimized in a phased approached in 2016, using the most up-to-date local data. We hope that in sharing Viet Nam’s experiences with ZIKV surveillance we can document what is often missing in the literature: the real-world challenges faced in public health and the practical solutions needed to conquer these obstacles.
